# Let Us Reason Together

**Published:** 1893-01

**Authors:** 


### Editorial.

### LET US REASON TOGETHER.

It is eminently proper at the beginning of a New Year to clear up the past and open a new account in the material things as well as in the moral and professional. This is the part of wisdom for all who desire orderly arrangement in the efforts to keep abreast with progress.

The year of 1893, it is anticipated, will comprise much within it for good or ill to dentistry. It is the year to celebrate the opening of the discovery of a new continent, and to the Columbian Exhibition all nations have been invited.

The celebration of the centennial of this republic did an enormous educational work. Never before were the people of the Old World so impressed with the developing power of the young nation as then. Never before had they appreciated the work accomplished, or did they come to understand the vast underlying possibilities in American life and energy. It did more than this, for it opened to the people who organized it the weak places in every branch of their own work. It changed, as never before, the thought and practice of a nation, and by its inspiriting influence has the average intellectual force been increased and strengthened in those things that make for higher development.

That this is true all who take a broad view of this educational period must acknowledge. It was more to American life than to those from whom that life originated.

If this view be accepted, how important must be the results of the present year! The vastness of the effort at Chicago almost paralyzes thought, yet mere size amounts to but little unless it combines with it the quality of superior excellence. It must show advancement. The people who come among us for the first time will expect to see the evidence of the active life of which they have heard so much, and those who are repeating the visit will expect to see evidence of growth. They will come here not to examine great collections, for America cannot hope to compete with those always existing in the Old World, but they do desire to examine the products of a younger civilization and learn the secret of that great prosperity, the origin of that wonderful inventive genius that has now for decades almost startled the world by its productive energy. If the Exhibition fails to meet this demand the country will be justly open to criticism that it has not kept pace with its previous record. This we know would not be true, and we believe that the Columbian Exhibition will demonstrate this as never before.

Dentistry was called upon to make its record there. The response from the organized associations was immediate and enthusiastic. The committees have been laboriously at work to carry out the wishes of those who placed them in these positions. The organization may be said to be finished. The officers have been selected, and now the completion of the work must devolve, in large degree, upon the profession as a whole.

In a former editorial we felt called upon to urge those who intended preparing papers to commence the work in earnest, and now we have a word for the lukewarm, the one who feels that he has not been recognized, or to him who may regard certain work as having been based on selfish motives.

Mistakes have been made, serious errors of judgment have been apparent, and many have been disappointed. Some have declared they would have no lot or part in it, and some have concluded that the World's Columbian Dental Congress could have no real scientific value. To these we have a word to say.

This hour is not one for personal bickering or the nursing of disappointed ambitions. It is the hour of self-abnegation for the good of the whole. It is strictly the devotional period, not in a religious sense, but in that sacrificing spirit that lays self on the altar built to commemorate professional progress.

Unity of thought, oneness of purpose, are essential, and we, therefore, deprecate anything that tends to diverse lines of action.

The World's Columbian Dental Congress is in the hands of the dentists of this country. They can make it more valuable than any previous convention if they so decide, but to do this there must be an untiring devotion to its interests. Activity is life, indifference means death, and we fear that the latter is the poison which may be doing more than open opposition. The past with its mistakes cannot be remedied, but the future can be so shaped by an energetic will that nothing will be desired to make the meeting a pronounced success.

Let all then come together with one purpose, and if criticism is to be made let it be reserved until the work has been accomplished and the end achieved.

